# Intracellular survival and vascular cell-to-cell transmission of *Porphyromonas gingivalis*

**DOI:** 10.1186/1471-2180-8-26

**Published:** 2008-02-06

**Authors:** Ling Li, Raynald Michel, Joshua Cohen, Arthur DeCarlo, Emil Kozarov

**Affiliations:** 1 Department of Biomedical Sciences, The Scripps Research Institute, Jupiter, FL 33458, USA; 2 Agenta Biotechnologies, Inc., Birmingham, AL 35211, USA; 3 NSU College of Dental Medicine, Fort Lauderdale, FL 33328, USA; 4 Center for Oral and Systemic Diseases, University of North Carolina, Chapel Hill, NC 27599-7455, USA

## Abstract

**Background:**

*Porphyromonas gingivalis *is associated with periodontal disease and invades different cell types including epithelial, endothelial and smooth muscle cells. In addition to *P. gingivalis *DNA, we have previously identified live invasive bacteria in atheromatous tissue. However, the mechanism of persistence of this organism in vascular tissues remains unclear. Therefore, the objective of this study was to analyze the ability of intracellular *P. gingivalis *to persist for extended periods of time, transmit to and possibly replicate in different cell types.

**Results:**

Using antibiotic protection assays, immunofluorescent and laser confocal microscopy, we found that after a prolonged intracellular phase, while *P. gingivalis *can still be detected by immunostaining, the intracellular organisms lose their ability to be recovered in vitro. Surprisingly however, intracellular *P. gingivalis *could be recovered in vitro upon co incubation with fresh vascular host cells. We then demonstrated that the organism was able to exit the initially infected host cells, then enter and multiply in new host cells. Further, we found that cell-to-cell contact increased the transmission rate but was not required for transmission. Finally, we found that the invasion of new host cells allowed *P. gingivalis *to increase its numbers.

**Conclusion:**

Our results suggest that the persistence of vascular tissue-embedded *P. gingivalis *is due to its ability to transmit among different cell types. This is the first communication demonstrating the intercellular transmission as a likely mechanism converting latent intracellular bacteria from state of dormancy to a viable state allowing for persistence of an inflammatory pathogen in vascular tissue.

## Background

*P. gingivalis*, a gram-negative anaerobe, plays a critical role in the development of adult periodontitis, a chronic inflammatory disease [1]. Epidemiological studies have demonstrated a positive association between periodontitis and cardiovascular diseases [2]. The immune response to this organism correlates with atherosclerosis [3,4]. We have detected intracellularly located *P. gingivalis *in periodontal tissues from patients [5]. We and others have also demonstrated the presence of DNA from periodontal pathogens such as *P. gingivalis *in atheromatous tissues [6,7]. Most importantly, we recently demonstrated for the first time the presence of viable invasive *P. gingivalis *in atheromatous plaque [8], thus implicating this chronic inflammatory agent in direct contribution to the development of inflammatory lesions at remote sites. This discovery led us to the legitimate question of the mechanism that would allow this organism to penetrate vascular walls upon dissemination and to persist in human vascular tissue.

Oral epithelia are likely the primary site for *P. gingivalis *infection but this bacterial species can enter the circulation following tooth brushing and other dental procedures therefore periodontitis is known to cause transient and low-grade bacteremia in patients [9,10]. This makes the periodontal site an "open gate" to circulation.

In addition to numerous other virulence factors, the invasion ability of *P. gingivalis *allows it to invade multiple cell types including animal cell lines, human vascular and gingival epithelial cell lines in vitro [11-17]. Tissue invasion is very likely a key virulence factor for this bacterium as it provides 1) a "privileged niche" with access to host protein (nutritional) and iron substrates, 2) a sequestration from the humoral and cellular immune response, and 3) a means for persistence that is essential for a chronic pathogen.

Studies from several groups have demonstrated the ability of *P. gingivalis *to invade gingival epithelial cells [11-13]. Studies of invasion in epithelial cells demonstrated that 1) the invasion is mediated by the interaction between *P. gingivalis *fimbriae and β1 integrin receptors [18]. 2) host cell cytoskeletal rearrangements are required for the entry [12,19]; 3) while inhibitors of protein kinase activity have no obvious effect on invasion, suppression of protease activity inhibited invasion process [12]; 4) during the first four hours of invasion, *P. gingivalis *appears to be able to replicate inside cells [12,13], but then recoverable CFU counts decrease [20]. In fact, we have detected intracellular *P. gingivalis *immunohistochemicallyin periodontal tissues from patients, located in the perinuclear region [5].

Many bacteria capable of invasion have been found to reside in phagosomes. Invasion of host cells is a common strategy for bacteria to escape immunosurvelliance and hostile environment. In phagocytic cells, *Mycobacterium tuberculosis *persists in phagosomes (review by [21]). It stops the normal maturation of phagosomes into an acidic, hydrolytic active environment, a phenomenon referred to as inhibition of phagosome-lysosome fusion [22]. Similarly, *Legionella pneumophila *enters its host, amoebae and protozoa [23] through phagocytosis, then rapidly modifies the phagosome to create an environment that supports its replication. Again a critical modification is the prevention of phagosome-lysosome fusion [24,25]. Virulent *Brucella abortus *also replicates in phagosomes with an increase in intraphagosomal pH [26]. The inhibition of the fusion between phagosomes and lysosomes seems to be a common theme to ensure the survival of invaded bacteria in phagosomes. When the inhibition is overcome, the acidification of the phagosomes eliminates the intracellular microbes [27,28]. In some cases, cytoplasmic bacteria can be ultimately trafficked into the lysosomal compartment for elimination [29,28].

*P. gingivalis *was found to be able to reside in phagosomes following invasion of endothelial cells [16] and possibly modify the phagosome to delay its fusion with lysosomes [30]. Therefore, host cell invasion by *P. gingivalis *may play a critical role in the development of vascular inflammatory lesions.

This raises the general question, what is the fate of this intracellular organism? Does *P. gingivalis *actually persist in tissues while in fact it is uncultivable? Even more important for vascular pathology, can *P. gingivalis *penetrate the vascular tissue, invading different cell types? The data on the intracellular fate of the organism itself hasn't clarified these broad questions yet while their answer is central for further elucidation of the persistent presence of this organism in atheromatous tissue and for clarification of the impact of that presence on the vascular wall.

Although there is an abundance of studies on *P. gingivalis *invasion of different cell lines [31], there is only one communication on *P. gingivalis *persistence that focuses on intercellular spreading [32]. The authors demonstrated that the bacteria can spread cell-to-cell, similarly to *Actinobacillus actinomycetemcomitans *[33]. However, the study is performed with gingival epithelial cells that are not representative for vascular pathology. Further, only one cell type was studied. Therefore, in the present study we decided to analyze the fate of intracellular *P. gingivalis *during extended incubation using cell-to-cell transmission among various cell types that exist in the vasculature *in vivo*. Using intracellular visualization by in situ immunofluorescent microscopy and bacterial recovery on blood agar plates (BAP), our experiments addressed the ability of *P. gingivalis *to survive over extended periods of time inside epithelial, endothelial (EC) and smooth muscle cells (SMC). In particular, we investigated whether *P. gingivalis *can leave the infected cells to invade and replicate in new host cells, including cells from different tissue type.

## Results

### *P. gingivalis *W83 invasion ability of KB, EC and SMC differs and can be increased by centrifugation

**Figure 1:**
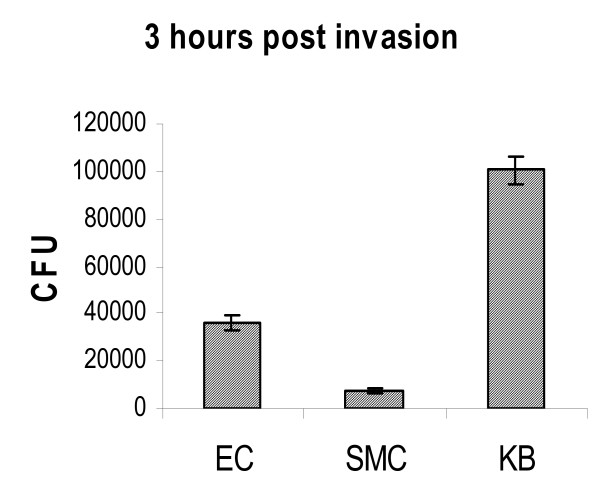
**Differential recovery of *P. gingivalis *W83 from different cell types on BAP at M.O.I. of 100**. The cells (10^5^per well) were seeded in 24-well plates 24 hours prior to invasion. Log-phase *P. gingivalis (*10^7^) were resuspended in 1.0 ml antibiotic-free medium and added to each well. Three hours post invasion, the cells were lysed, *P. gingivalis *was recovered and CFU were enumerated. Data presented are average of four independent experiments, four plates per experiment. EC, primary endothelial cells; SMC, primary smooth muscle cells; KB, continuous epithelial cell line. Error bars denote standard deviation.

**Figure 2:**
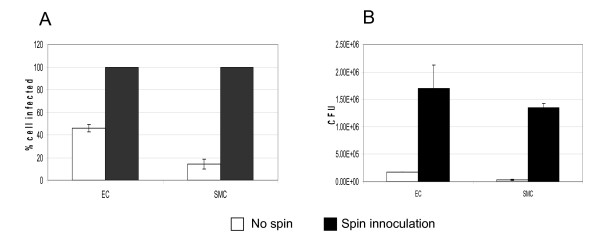
**Spin-inoculation increases *P. gingivalis *W83 invasion of EC and SMC at M.O.I. of 100**. For spin inoculation, after the addition of the bacteria, the plate was centrifuged at 1000 × g for 10 minutes at room temperature. Three hours post invasion, the intracellular *P. gingivalis *was enumerated by immunofluorescent staining (2A) and recovered on BAP (2B). For immunofluorescent staining, five fields were viewed and the percentage of *P. gingivalis*-containing cells was calculated. Means and standard deviations are derived from five different fields. For CFU on BAP, the means and standard deviations were derived from four separate experiments.

To investigate the ability of *P. gingivalis *to invade and survive inside epithelial, endothelial and smooth muscle cells, we first assessed the invasion ability of *P. gingivalis*W83 to epithelial (KB) cells, EC and SMC at multiplicity of infection (M.O.I.) of 100 using an antibiotic protection assay as described [16]. At three hours post invasion, the host cells were lysed and the number of invaded *P. gingivalis *was enumerated on BAP. As shown in Figure 1, the number of *P. gingivalis *colony-forming units (CFU) recovered on BAP varied among cell lines, suggesting differential invasion ability, as previously observed [14]. Among these three cell lines, the *P. gingivalis *recovery from KB on BAP was the highest while the recovery from SMC was the lowest. This may be due to the differential affinity between *P. gingivalis *and each cell type. This differential affinity could be overcome by spin inoculation. When increasing *P*.*gingivalis *and cell contact by spin inoculation (1000 × g, 10 minutes at room temperature), the bacterial invasion into each cell type increased by orders of magnitude (Figure 2).

### *P. gingivalis *W83 invasion doesn't affect the viability of the infected host cells

Since infection using the spin inoculation protocol can cause considerable stress to the host cells, we verified their viability using MTT [3-(4,5-dimethylthiazol-2-yl)-2,5-diphenyltetrazolium bromide] cell proliferation assay. Ten thousand cells were seeded per well in a 96-well format, reaching 70% confluency, similarly to the confluency we used in the invasion assays. MTT assay measures the ability of living cells to convert yellow water soluble MTT into insoluble purple formazan. With uninfected cells as a control, *P. gingivalis *invasion at MOI of 100 with spin inoculation has no effect on cell proliferation/metabolism in all three cell types using MTT assays at 3 hr, 24 hr and 48 hr post infection (data not shown).

To independently confirm the result of the MTT assays, invasion assays were also carried out where the host cells were counted using a hemocytometer at each time point (3 hr, 24 hr and 48 hr post invasion). Similarly, there was no difference in cell numbers between invaded cells and uninfected controls in all three cell types (data not shown).

### Recovery of intracellular *P. gingivalis *W83 on blood agar plates (BAP) declines over time after invasion

**Figure 3:**
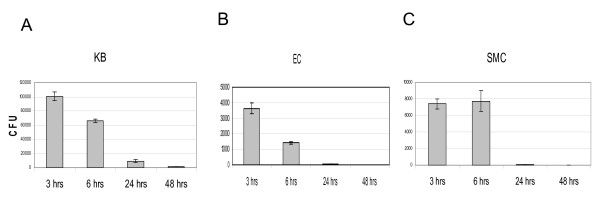
**Invasion kinetics of *P. gingivalis *W83**. The cells were infected at M.O.I of 100 without spin inoculation. At each time point *P. gingivalis *were collected, plated on BAP and CFU were enumerated. Means and standard deviations were derived from four separate experiments involving four plates for each experiment. A, KB cells; B, EC; C, SMC.

In order to study the replication kinetics of the intracellular *P. gingivalis*, we harvested intracellular *P. gingivalis *using the antibiotic protection assay at various time points post invasion. To our surprise, as shown in Figure 3, we did not detect intracellular *P. gingivalis *amplification using the antibiotic protection assay at the described infection condition. Instead, a sharp decline in the number of *P. gingivalis *recovered on BAP at 24 hr post invasion was detected and no bacteria could be recovered at 48 hour time point from EC and SMC (only very low numbers of *P. gingivalis *could be recovered from KB cells). These experiments were repeated five times to confirm the unexpected data. When facilitating the invasion by spin inoculation, the number of *P. gingivalis *recovered on BAP at 3 hr time point increased 10 fold as described above (Figure 2B), but still no *P. gingivalis *could be recovered on BAP from EC and SMC at 48 hr post invasion. Similarly, only very low number of *P. gingivalis *was recovered from KB cells at 48 hr time point (Table 1, "medium only" row).

**Table 1:** Number of CFU recovered on BAP after co-culturing infected cells with fresh host cells

**Addition of new host cells**		**Number of CFU recovered on BAP after 24 hour co-culture**		
		
		**iEC**	**iSMA**	**iKB**
Co-culture	Medium only	0	0	33 +/- 14
	KB	72 +/- 8	400 +/- 12	2169 +/- 107
	SMC	26 +/- 6	210 +/- 18	191 +/- 3
Transwell System	Medium only	0	0	0
	KB	7 +/- 3	64 +/- 11	361 +/- 19
	SMC	2 +/- 1	30 +/- 5	79 +/- 15

Values are means +/- standard deviation (three samples per group) for the number of colony forming unit (CFU) recovered on blood agar plates (BAP). Multiplicity of infection (M.O.I.), 100.

### In contrast to the recovery rate on BAP, immunofluorescent microscopy demonstrates the presence of a large number of *P. gingivalis *at 48 hour post invasion

**Figure 4:**
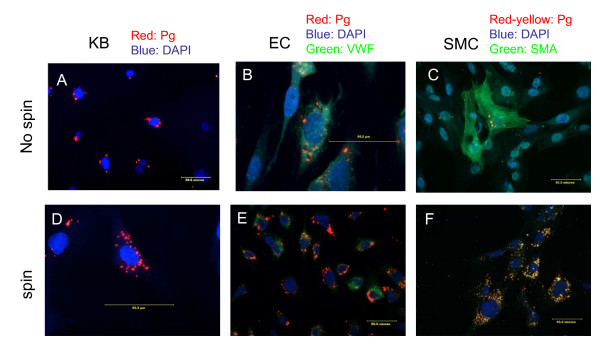
**Immunofluorescent images of *P. gingivalis *in KB, EC and SMC 48 hours post-invasion (M.O.I. of 100)**. Micrographs A, B and C represent infected KB, EC and SMC, respectively. Micrographs D, E, and F represent infected KB, EC and SMC using the spin inoculation protocol. Scale bar is 50.0 μm. For images C and F, murine monoclonal anti-*P. gingivalis *antibody, 61BG1.3 was used followed by Cy3-labeled secondary antibodies. For smooth muscle actin (SMA), murine monoclonal anti-SMA antibody was used followed by FITC labeled secondary antibodies. Since both Cy3- and FITC-labeled secondary antibodies stained murine monoclonal antibodies, *P. gingivalis *may appear yellow. VWF, von Willebrand factor.

Surprised by the loss of *P. gingivalis *recovery over time on BAP, we performed immunofluorescent microscopy to verify the status of intracellular *P. gingivalis. P. gingivalis *was easily found throughout the cultured cells at each of the time points, including the 48-hr time point. As demonstrated in Figure 4, at 48 hr post-invasion, while no *P. gingivalis *can be retrieved on BAP, the bacteria could be observed with the immunofluorescent staining and most were perinuclear in all three cell types. This is consistent with the observation made in primary oral epithelial cells [34]. Similarly, under the condition of spin inoculation, while no *P. gingvalis *was recovered on BAP, copious numbers of the intracellular *P. gingivalis *could be found by immunofluorescent staining (Figure 4).

### *P. gingivalis *is able to transmit from infected to uninfected cells

Once we found that no *P. gingivalis *could be recovered on BAP from EC and SMC at 48 hours post invasion, we investigated the possibility of intracellular *P. gingivalis *leaving the host cells to invade and possibly replicate in new host cells.

Endothelial, SMC, and KB cells were infected with *P. gingivalis *at MOI of 100 using the spin inoculation protocol. Twenty-four hours post invasion, the invaded host cells were harvested by trypsinization, 2 × 10^4 ^of these initially infected cells were mixed with 8 × 10^4 ^fresh uninfected host cells and plated in 24 well plates using the uninfected host cell culture media. After twenty-four hours of co culturing (48 hours post initial invasion), the cells from each well were lysed by sterile water and plated on BAP. The results are shown in Table 1 (upper portion). Surprisingly, the amount of colony forming units (CFU) recovered on BAP increased above the zero or minimal recovery obtained after 48 hour post-infection in the initial cultures alone, indicating that *P. gingivalis *had transmitted and possibly replicated in the new host cells.

**Figure 5:**
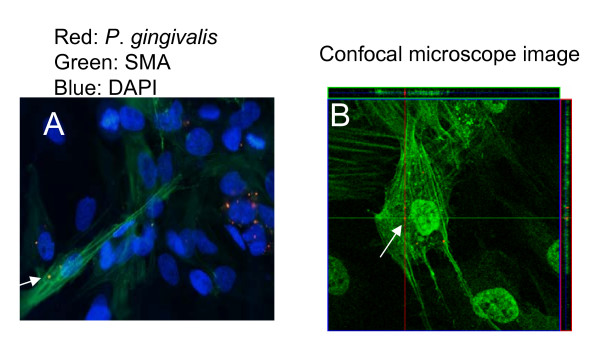
***P. gingivalis *can be localized in newly added host cells**. (A) 24 hour co-culture of primary invaded endothelial cell (iEC) with freshly added SMC. The arrow identifies the presence of *P. gingivalis *in SMC. (B) The confocal microscope image (depth of 1.57 μm) identifying *P. gingivalis *internalized by a smooth muscle cell after co-culturing with invaded endothelial cells.

To confirm that the CFU counts are due to *P. gingivalis *transmitted into new host cells, we further performed immunofluorescent staining using SMC as new host cells. Smooth muscle cells were selected in order to utilize anti-SMC actin antibody that is able to stain intracellularly, therefore can be used to determine if the bacteria are intracellular. As described above, 2 × 10^4 ^*P. gingivalis*-invaded EC (iEC) were co cultured with 8 × 10^4 ^uninfected SMC cells. Twenty-four hours later (48 hours post initial invasion), the cells were fixed and stained using antibodies against *P. gingivalis *and SMC actin. The result indicated presence of *P. gingivalis *in the SMC cells by both immunofluorescent (Figure 5A) and confocal (Figure 5B) microscope analysis, confirming our hypothesis that *P. gingivalis *is able to leave primary host and enter new host cells in the co culture system (Figure 5 and Table 1).

### *P. gingivalis *is able to exit the primary host cells, to infiltrate the medium and to invade new host cells. The cell-cell contact is not required for bacterial cell-to-cell transmission

**Figure 6:**
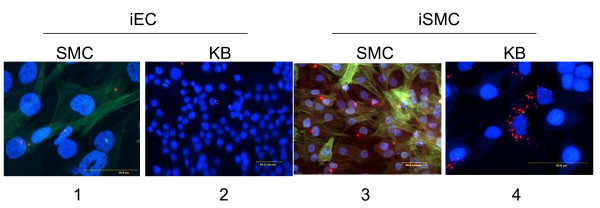
***P. gingivalis *transmission into new host cells does not require cell-to-cell contact**. This is demonstrated using the Transwell inserts to separate initially infected from subsequently added host cells. The initial infected cells were spin inoculated at M.O.I. of 100. Twenty-four hours later, 2 × 10^4 ^invaded cells were harvested and seeded into a Transwell insert. The insert was placed on top of 8 × 10^4 ^uninfected cells. Following combinations were tested. 1) Transmission from the insert with infected EC (iEC) to SMC at the bottom compartment; 2) Transmission from iEC to KB cells; 3) Transmission from iSMC to SMC; and 4) Transmission from iSMC to KB. After 24 hour incubation with the Transwell insert, the cells at the bottom were fixed. For *P. gingivalis*, anti-*P. gingivalis *antibody, 61BG1.3 was used, followed by Cy3-labeled secondary antibody. For SMC, anti-smooth muscle actin antibody was used followed by FITC-labeled secondary antibody. Blue, DAPI staining. Red, Pg; Blue, DAPI; Green, SMA. Scale bar is 50 μm.

To determine whether cell-cell contact is required for the *P. gingivalis *transmission to new host cells, a Transwell system was utilized to separate the initially infected host cell from uninfected new host cells as described above. The following host cell combinations were tested: 1) the Transwell insert containing infected EC (iEC) placed on top of uninfected SMC; 2) similar insert placed on top of uninfected KB cells; 3) the insert containing iSMC placed on top of uninfected SMC; 4, similar insert placed on top of uninfected KB cells. After 24-hour incubation with the Transwell insert, the cells at the bottom were fixed and stained by anti-*P. gingivalis *monoclonal antibody followed by Cy3-labeled secondary antibodies and also with anti-SMA antibody followed by FITC labeled secondary antibodies. DAPI staining was used to visualize the nuclei as before (Figure 6). Despite the physical barrier between the two cell layers, the bacteria were found in the new host cells, indicating that *P. gingivalis *can exit primarily infected host cells, penetrate the media and invade new host cells.

### The cell contact between infected cells and new host cells increases the transmission rate

Furthermore, after the removal of Transwell inserts, we also lysed the cells at the bottom compartment by sterile H_2_O and plated the lysate on BAP. In concordance with the microscopy observations, *P. gingivalis *could be recovered from cells that had no direct contact with infected cells (Table 1, bottom). However, based on the number of *P. gingivalis *CFU recovered on the plates, the cell contact between infected cells and new host cells increases the transmission rate.

## Discussion

Periodontitis, one of the most prevalent infectious diseases [35] is caused by a small number of bacteria, including *P. gingivalis *that we have shown to reside in gingival tissue from periodontal patients [5] and, even more significantly, in atheromatous plaque [8]. The main challenge for tissue-embedded *P. gingivalis *is to survive and we set out to explore a possible mechanism of its persistence.

Here we present data demonstrating that in our experimental set-up *P. gingivalis *strain W83 displays an invasive ability that differs by up to an order of magnitude in the tested cell lines and that this difference could be minimized by spin inoculation. The differential invasion efficiency observed for different cell types is likely due to different interactions between *P. gingivalis *and the types of cell surface receptors present on the surface of the different cell types that are involved in the invasion process. *P. gingivalis *fimbriae and hemagglutinating adhesins have been reported to serve as ligands for the initial attachment to the host cells [36,37]. Beta integrins [18], CD14 and the β2 integrins CD11b/CD182 [38] have been reported to be host cell receptors for *P. gingivalis *fimbriae. Achieving higher CFU numbers after spin-inoculation of the tested cell lines is likely due to the increased contact between these *P. gingivalis *ligands and the host surface receptors.

We also demonstrate that the 48-hr invasion (at M.O.I. of 100) of the studied three different cell types, KB, EC and SMC doesn't affect the viability of the host cells, in contrast to the data for gingival fibroblasts [39]. Again, this observation is most likely due to the different cell type, and in terms of invasion of primary EC is supported by a recent report [30]. This effect is plausibly due to the advantage for the bacterium to prevent the death of the host, thus evading the humoral or cellular immune response and securing a source of nutrients.

We next found that *P. gingivalis *could be detected intracellularly at 48 hr post invasion in large numbers by immunofluorescent microscopy. While different entry pathways and intracellular trafficking in various cell types have been proposed [40-42,16,11] we observed that at a relatively high M.O.I. of 100, virtually all host cells in culture contained several microbes, and that recovery of viable intracellular *P. gingivalis *was relatively high a few hours post-invasion, which was also expected. Over a period of 48 hr, however, while the level of intracellular localization did not change, the recovery of viable microbes on BAP diminished to virtually zero for SMC and EC (and very low numbers for KB). Most of the bacteria in all three cell types are perinuclear at this stage, similarly to what has been demonstrated in primary oral epithelial cells [34,19].

It is unclear why *P. gingivalis *could not be recovered on BAP at this stage. The inability to recover *P. gingivalis *on BAP has been reported before. For example, we found high amount of *P. gingivalis *genomic DNA in atheromas using Q-PCR [7] but no bacteria could be directly recovered on blood agar plates, as previously observed [8,43]. One of the possibilities is that *P. gingivalis *may not survive the intracellular environment. However, based on our previous data [8] and on the data presented in this study it can not be ruled out that *P. gingivalis *may actually enter a latent state to reside intracellularly, resulting in inability to be cultivated on BAP, and that it may leave such a state upon contact with fresh host cells.

Therefore, there are two possible scenarios with these observations. The first scenario is that some of sequestered intracellular *P. gingivalis *had become non-viable. Several killing mechanisms may be proposed, for example the bacteria may be killed by trafficking into the phagolysosomal or autolysosomal compartment [30]. The trigger for this to occur could be initiated by a high number of intracellular *P. gingivalis *or by depletion of intracellular nutrients. Such mechanism may be used to control the *P. gingivalis *population. Previously, another *P. gingivalis *strain (FDC381) has been reported to persist in KB cells for eight days [13]. Using the antibiotic protection assay, the amount of *P. gingivalis *FDC381 recovered on BAP from KB cells started to decline at day 2. However, in that case, KB was infected at a M.O.I. of 2, or 50-fold less than what we used in our experimental system. This therefore is in line with the hypothesis that the number of intracellular *P. gingivalis *may serve as a trigger to trafficking to a lysosomal compartment thus regulating the number of intracellular *P. gingivalis*.

However, this hypothesis does not rule out another possible scenario, that *P. gingivalis *may not be killed by host cells and that number of bacteria may be instead converted into a stage of dormancy. Such hypothesis, supported by ours and others' data, is that the cell-to-cell transmission converts latent intracellular bacteria from state of dormancy to a cultivable state. It explains the observations that dormant intracellular *P. gingivalis *could not be recovered in vitro by plating, but only after transmission to fresh host cells. In addition, further support of this hypothesis originates from the global genomic profile data of intracellular *P. gingivalis *[40]. Using microarray genomics, that study demonstrated that the majority of differentially regulated *P. gingivalis *genes (52 of 63) were down-regulated upon invasion. Even more interesting, a substantial number of the down-regulated genes (21 of 52) were involved in protein synthesis, transcription, and energy metabolism. Such specificity in the down regulation indicates a reduced growth rate of intracellular bacteria, or a latent state.

Our hypothesis that the cell-to-cell transmission converts latent intracellular bacteria from state of dormancy to a cultivable state was first made possible based on our previous data on identification of viable *P. gingivalis *in atherosclerotic tissue only after transfer to new host via co incubation with fresh cell culture [8]. Subsequently, cell-cell transfer of *P. gingivalis *has been demonstrated within oral epithelial cells as well [32]. Further confirming our observations of leaving the primary infected cell, extracelullar *P. gingivalis *has been detected in invaded KB culture supernatants [20]. In the present study, this hypothesis is further supported by our experimental data demonstrating that *P. gingivalis *can spread intercellularly between the same as well as between different cell types, as a consequence substantially increasing the CFU count number when recovered in vitro. This cell-cell transmission took place even when the new cells were separated from the already invaded cells via Transwell set-up, although the numbers were 6–10 times lower, apparently due to the separation (Table 1). Finally, we demonstrated that cell contact between infected cells and new host cells increases the rate of transmission.

The mechanism of this transmission remains to be determined, but bacterial replication in the medium is unlikely for this strict anaerobe, especially providing its average replication time (5–6 hrs) and the rapid internalization by host cells (~10 min). In addition, we tested whether *P. gingivalis *can survive and replicate in media for up to 48 hours and the results were negative (data not shown). To differentiate between these two scenarios, intracellular trafficking, viability of intercellular *P. gingivalis *and its gene expression profile at 48 hours post-invasion in different cell types must be extensively studied in parallel.

## Conclusion

In summary, here we present data demonstrating that in our experimental set-up *P. gingivalis *strain W83: (1) displays an invasive ability that differs by up to an order of magnitude in the tested cell lines and the difference could be minimized by spin inoculation, (2) could be detected intracellularly in large numbers at 48 hr post invasion by immunofluorescent microscopy, but most of the bacteria could not be recovered on BAP; (3) can spread intercellularly between the same as well as between different cell types; (4) the invasion of fresh host cells ends the dormant, uncultivable state allowing for *P. gingivalis *isolation in vitro, and (5) cell-cell contact between infected cells and new host cells increases the rate of transmission.

Therefore, our working model, supported by our observations is as follows. Upon invasion, *P. gingivalis *is able to reside/replicate inside the host cells for a limited time. A high number of *P. gingivalis *or the depletion of nutrients may then initiate the killing (or turning into a dormant stage) of *P. gingivalis *leading to control of bacterial population numbers, for example by trafficking it to the lysosomal compartments. In addition, facing hostile environment, some *P. gingivalis *would exit into intracellular space and invade new host cell, thus escaping from the dormant stage, further penetrating the vascular tissue transcellularly and becoming both invasive and cultivable in vitro.

Using such mechanism, *P. gingivalis *would control its population in vascular tissues yet allow for a persistent infection, similarly to infection and spreading among primary gingival epithelial cells [32]. With the epithelial host, there was a very low level of cell-to-cell transmission during the early stage of invasion (3 hr time point), but the rate of transmission increased substantially at later stages of infection (24 hr and 48 hr time point). The increased transmission rate at the late stage of infection is in agreement with our hypothesis of cell-cell transmission-mediated bacterial survival and persistence. Our observations support a model of atherosclerosis where the inflammation is initiated and/or exacerbated by invasive inflammatory agents that may endure intracellularly in a dormant stage, whose numbers are controlled but that can also escape the host, become invasive and infect new host cells thus persisting in the vascular tissue. This model is currently under further investigation. Studies of the molecular machinery of *P. gingivalis *cell-cell transmission will further elucidate the mechanisms by which *P. gingivalis *is able to establish persistent infection of vascular walls and ultimately contribute to vascular inflammations.

## Methods

***Porphyromonas gingivalis *strain W83 **was grown anaerobically in Bacto™ Tryptic Soy Broth (BD biosciences, San Jose, CA) supplemented with 0.5% yeast extract, 0.05% L-cysteine, hemin (0.05 mg/ml), and vitamin K1 (0.1 mg/ml). Blood agar plates (BAP) were made as described [16].

**KB **(ATCC CCL-17 HeLa), an epithelial squamous cell carcinoma line, was maintained in Dulbecco's minimum essential medium (DMEM high glucose) with 10% FBS, 2 mM Glutamine and 25 mM HEPES, PH 7.5 (Invitrogen, Carlsbad, CA). The culture medium was supplemented with 100 units/ml of penicillin, 100 μg/ml of streptomycin, and 0.25 μg/ml of amphotericin B (Invitrogen, Carlsbad, CA).

### Endothelial cell isolation and culture conditions

The primary endothelial cells (EC) were provided by Hong Yu (University of Miami) and grown as described [44]. EC were isolated as follows. Human saphenous veins left over from bypass surgery were harvested and transported to the laboratory in Hanks Balanced Salt Solution (HBSS) containing 25 mM HEPES (pH 7.5), ampicillin (200 U/ml), streptomycin (200 μg/ml), kanamycin (100 μg/ml) and amphotericin B (1.25 μg/ml). Veins were flushed with the buffer, clamped at one end, filled with 0.05% collagenase I in Dulbecco's phosphate buffered saline (DPBS) and incubated for 15 minutes at 37°C. Cells were pelleted at 200 × g for 10 minutes at room temperature. The cells were then suspended and maintained in MCDB 131 medium (Invitrogen, Carlsbad, CA) supplemented with 2 mM glutamine, 20% fetal bovine serum (FBS), 50 μg/ml heparin, 50 μg/ml endothelial cell growth supplement (BD Biosicences, San Jose, CA), 100 units/ml penicillin, 100 μg/ml streptomycin, and 0.25 μg/ml amphotericin B (Invitrogen, Carlsbad, CA). The EC were used between passage 8 and 15 and positively stained for von Willebrand factor (VWF) and factor VIII. Control staining with anti-smooth muscle actin (SMA) antibody and anti-*P. gingivalis *antibody was negative (data not shown). The isolation protocol was approved by the Institutional Review Board at University of Miami.

### Smooth muscle cell isolation and culture conditions

The primary smooth muscle cells (SMC) were also isolated from human saphenous veins and provided by Hong Yu (University of Miami). The protocol was also approved by Institutional Review Board at University of Miami. To isolate SMC, the intimal endothelial layer of harvested vein was scraped off with a sterile blade. The resultant inner media, which contained almost exclusively vascular smooth muscle, was minced and incubated in 10 ml of a collagenase (1.8 mg/ml, Sigma, St. Louis, Mo) and elastase (0.2 mg/ml, Sigma) solution for 1.5 hours at 37°C. The resulting single-cell suspensions were then removed and pelleted by centrifugation at 200 × g for 10 minutes. The cells were then suspended and maintained in William E (Invitrogen) containing 20% FBS, 100 units/ml penicillin, 100 μg/ml streptomycin, and 0.25 μg/ml amphotericin B. The SMC can be grown up to passage 20 and still expresse smooth muscle actin. Control staining with anti-VWF antibody and anti-*P. gingivalis *antibody was negative (data not shown).

### Antibiotic protection assay

The antibiotic protection assay was performed as described in [16]. In short, 10^5 ^cells were seeded per well in 24-well plates. Next day, the cell medium was removed, and 10^7 ^log-phase *P. gingivalis *suspended in 1.0 ml antibiotic-free cell culture medium were added (MOI of 100) and the cells were incubated for 90 minutes at 37°C, 5% CO_2_. The number of *P. gingivalis *was determined spectrophotometrically. For spin inoculation, the 24-well plate was spun at 1000 × g for 10 minutes prior to the 90-minute incubation. The cells were then washed gently three times with HBSS (Invitrogen, Carlsbad, Calif.) and media containing gentamicin (300 μg/ml) and metronidazole (200 μg/ml) was added followed by incubation for another 60 minutes to kill extracellular *P. gingivalis*. The cells were then washed again three times with HBSS and maintained in their culture medium. At each time point (3 hours, 24 hours and 48 hours after the initial contact between the bacteria and host cells), 1 ml sterile ddH_2_O was added and cells were lysed for 20 minutes at room temperature. The lysate was collected and *P. gingivalis *was pelleted at 16,000 × g for 2 minutes before plating in quadruplicate on blood agar plates (BAP).

### Cell proliferation assays

The cell proliferation assays were performed using a Vybrant MTT cell proliferation assay kit (Invitrogen, Carlsbad, Calif.) and cell counting. The MTT assay was performed in a 96 well format according to manufacturer's recommendation. Ten-thousand cells were seeded per well, reaching 70% confluency and then 1 × 10^6 ^*P. gingivalis *in 100 μl culture media were added per well. The plate was spun at 1000 × g for 10 minutes and incubated at 37°C, 5% CO_2 _for 90 minutes. After gentamicin (300 μg/ml) and metronidazole (200 μg/ml) treatment, the cells were washed with HBSS and maintained in their culture medium. At each time point, the medium was replaced with 100 μl fresh medium, 10 μl of 12 mM MTT 3-(4,5-dimethylthiazol-2-yl)-2,5-diphenyltetrazolium bromide stock solution was added to each well and cells were incubated for 4 hours at 37°C. The reaction was terminated by the addition of 100 μl of SDS.HCl solution and further incubation for 12 hours at 37°C. Finally, each sample was mixed and absorbance at 570 nm was measured.

For manual counting, 1 × 10^5 ^cells were seeded per well in 24-well plates and infected with *P. gingivalis *at MOI of 100 with or without spin inoculation. After gentamicin (300 μg/ml) and metronidazole (200 μg/ml) treatment to kill the extracelullar bacteria, the cells were washed with HBSS and maintained in their culture media. At each time point, cells were trypsinized, pelleted and suspended in 10 μl tissue culture medium. The cells were then mixed with trypan blue and counted using a hemocytometer.

### Co-culture assays

The co culture assays were performed in two formats. For the first format, the infected cells were mixed with uninfected cells at ratio 1:4. Briefly, 24 hours after the initial infection, 2 × 10^4 ^infected cells, harvested by trypsinization, were mixed with 8 × 10^4 ^uninfected cells and the mixture was transferred into a fresh well of a 24-well plate using the uninfected cell culture medium. There was no additional antibiotic except the standard Pen-Strep-Amphotericin B present in the tissue culture media as described above. After 24 hours co-culture, the number of intracellular *P. gingivalis *was either determined by host cell lysis and plating on BAP or by in situ immunofluorescent staining. Alternatively, a Transwell system from Corning (Acton, Mass.) was utilized to separate the primary host cell from new host cells during co-culture. For that, 2 × 10^4 ^invaded cells (24 hours post invasion) were harvested and seeded into a 6.5-mm diameter, 3-μm pore size Transwell insert. The inserts were then placed on top of 8 × 10^4 ^cells per well in 24 well plates. The invaded cells and the uninfected cells were cultured in their corresponding media. Twenty-four hours later, the Transwell inserts were discarded and cells at the bottom compartment were either lysed for plating on BAP or fixed for the immunofluorescent staining. For that, cells were seeded on cover slips as described in "Immunofluorescent and laser confocal microscopy" section.

### Antibodies

Murine monoclonal anti-human von Willebrand's factor (VWF) antibody, rabbit polyclonal anti-human VWF antibody and murine monoclonal anti-human smooth muscle actin (SMA) antibody were purchased from Dakocytomation (Glostrup, Denmark). Rabbit polyclonal anti-human SMA antibody was obtained from Abcam (Cambridge, Mass.). Goat anti-human factor VIII-related antigen antibody was purchased from Atlantic Antibodies (Scarborough, Maine). Murine monoclonal anti-*P. gingivalis *hemagglutinin A antibody 61BG1.3 [45] was a gift from Rudolf Gmür (Zürich, Switzerland). FITC- or Cy3-labeled anti-mouse antibody and FITC- or Cy3-labeled anti-rabbit antibody were from Jackson Immunolaboratory (West Grove, Penn.).

### Immunofluorescent and laser confocal microscopy

Cells, especially endothelial cells, adhered and grew better when the cover slip was coated with collagen type I or IV. We chose to use both collagens to maximize our cell adherent and proliferation rate. Briefly, 12-mm round cover slips pretreated with collagen type IV and collagen type I (BD) were placed into 24 well plates with one coverslip per well, 1 × 10^5 ^cells were then seeded per well and *P. gingivalis *W83 suspension was added at M.O.I.~100 with or without spin inoculation. At each time point, the cells were fixed by 4% paraformaldehyde in PBS at room temperature for 30 minutes and permeabilized by ice-cold methanol for 5 minutes at -20°C. To minimize the background, the cells were further incubated in blocking solution (5% BSA, 0.1% Tween 20 in PBS) for 30 minutes at room temperature. Primary antibodies were applied in the blocking solution for one hour at room temperature as follows: monoclonal mouse anti-*P. gingivalis *hemagglutinin A antibody 61BG1.3, 1:50; polyclonal rabbit anti-VWF antibody, 1:100 dilution; monoclonal anti-α SMA antibody,1:100; polyclonal rabbit anti-α SMA antibody, 1:100. After the incubation, the cells were washed three times with PBS and incubated with diluted 1:100 fluorescently labeled secondary antibodies in the blocking solution at room temperature for one hour in dark. After washing with PBS, DAPI (1 μg/ml in PBS) was added and the cells were incubated for additional 20 minutes at room temperature to stain the nuclei. The cells were then washed again three times with PBS, the cover slip was mounted on a glass slide with gel mount (Biomeda, Foster City, Calif.) and sealed with nail polish. Control staining with anti-*P. gingivalis *61BG1.3 antibody was carried out for all cell lines for each experiment with consistently negative results (data not shown). No *P. gingivalis *was detected in these cell lines prior to being infected. Images were acquired with a Leica DML fluorescent microscope equipped with a CCD cooled camera and Advanced Spot Imaging System and with Zeiss confocal microscope.

## Authors' contributions

LL, RM and JC carried out the immunoassays. LL, AD and EK participated in the design of the study. LL conceived the study, and helped to draft the manuscript. All authors read and approved the final manuscript.
